# Evaluating Japanese language learning strategies through learner and expert perspectives: a fuzzy multi-criteria decision-making approach

**DOI:** 10.3389/fpsyg.2026.1902458

**Published:** 2026-08-21

**Authors:** Lin Liao, Qiqiang Zhou, Nobuharu Sato

**Affiliations:** 1 School of Foreign Studies, Hunan University of Science and Technology, Xiangtan, Hunan, China; 2 Graduate School of Humanities and Social Sciences, Hiroshima University, Hiroshima, Japan

**Keywords:** educational evaluation, fuzzy multi-criteria decision making, instructional design, Japanese language learning, learner motivation, second language acquisition

## Abstract

**Background:**

Evaluating Japanese language learning strategies is challenging because language acquisition depends on cognitive, motivational, instructional, and sociocultural factors that cannot be adequately assessed using conventional evaluation methods. A systematic framework that integrates multiple educational dimensions and accommodates uncertainty is therefore needed.

**Methods:**

This study proposes a hybrid Fuzzy Analytic Hierarchy Process (FAHP) and Fuzzy Technique for Order of Preference by Similarity to Ideal Solution (FTOPSIS) framework to evaluate Japanese language learning strategies from expert and learner perspectives. Six evaluation criteria were identified through a comprehensive literature review and expert consultation. Expert judgments were used to derive criterion weights using FAHP, while data collected from 30 Japanese language learners representing different age groups and proficiency levels were incorporated into the FTOPSIS model to rank alternative learning strategies. A sensitivity analysis was conducted to evaluate the robustness of the proposed framework.

**Results:**

Linguistic Complexity (22.1%), Cultural Immersion (20.4%), and Learner Motivation (17.8%) were identified as the most influential evaluation criteria. Among the evaluated strategies, the Integrated Immersive Strategy achieved the highest closeness coefficient (0.837), indicating the strongest alignment with the ideal learning profile. Sensitivity analysis confirmed that the ranking remained stable under substantial variations in criterion weights, demonstrating the reliability of the proposed model.

**Conclusion:**

The proposed FAHP–FTOPSIS framework provides a transparent and robust approach to evaluating Japanese language learning strategies by integrating expert knowledge with learner perspectives under uncertainty. The findings support the educational value of immersive, learner-centered, and technology-supported instructional approaches and provide practical guidance for educators, curriculum designers, and researchers seeking evidence-based strategies for Japanese language education.

## 1 Introduction

Japanese language education has expanded considerably over the past two decades due to increased academic exchange, economic cooperation, and cultural interest in Japan. The widespread adoption of digital learning platforms, blended instruction, and learner-centered pedagogies has transformed how Japanese is taught and learned (Kayashima et al., 2022; Aarthi et al., 2026). While traditional approaches emphasized grammar translation and memorization, contemporary instruction increasingly promotes communicative competence, cultural understanding, technology-supported learning, and learner autonomy (Zhang et al., 2025; Sun et al., 2025). These developments have improved learning opportunities but have also increased the complexity of evaluating the effectiveness of different instructional strategies (Ota, 2018; Shao et al., 2025).

These developments are consistent with the principles of Second Language Acquisition (SLA), which emphasize that language learning is influenced by cognitive, motivational, instructional, and sociocultural factors. Contemporary SLA research recognizes that successful language acquisition depends not only on linguistic knowledge but also on learner engagement, authentic communication, meaningful cultural exposure, and supportive learning environments (Saville-Troike and Barto, 2016; Ellis, 2004). Consequently, evaluating Japanese language learning strategies requires a framework that integrates these multiple dimensions.

Japanese language education plays a vital role in learners' intellectual and intercultural development by fostering linguistic competence, cognitive flexibility, and global awareness (Sakamoto and Roger, 2023; Goh et al., 2025). Japanese has become increasingly popular among second-language learners due to Japan's prominent influence in technology, pop culture, and international business (Islam, 2024; Dizon, 2021). Beyond its cultural appeal, the language demands a high level of analytical and memorization skills, especially due to the complexity of its writing systems, kanji, hiragana, and katakana, as well as its nuanced honorific expressions and contextual grammar rules (Rossides, 2025; Robertson, 2020).

However, assessing the effectiveness of Japanese language learning strategies remains a major challenge. Learner outcomes are influenced by numerous factors, including instructional methodology, linguistic difficulty, learner motivation, cultural immersion, and access to learning resources (Huang et al., 2020; Bower, 2019; Wang et al., 2026). Conventional evaluation methods rely heavily on test scores or anecdotal feedback, making it difficult to objectively compare learning approaches (Liu et al., 2026; Zhang and Chen, 2026). Moreover, the absence of a standardized assessment model prevents educators from systematically improving instruction or tailoring strategies to individual needs (Csapó and Molnár, 2019; Alamri et al., 2021; Abujaber et al., 2023). To address these challenges, this study develops a learner-centered evaluation framework that integrates expert judgments and learner perspectives to assess the effectiveness of Japanese language learning strategies across multiple educational dimensions.

Evaluating the success of Japanese-language learning strategies poses substantial challenges due to the multidimensional nature of language acquisition. Unlike subjects with standardized outputs, such as mathematics or science, language learning involves both measurable competencies, such as vocabulary size and reading comprehension, and less tangible factors, such as cultural understanding, pragmatic fluency, and learner identity (Tu et al., 2026; Zhu et al., 2026; Feng et al., 2025). Several issues complicate evaluation efforts, including the subjectivity of language proficiency assessments, variability in learners' backgrounds, and the interplay among cognitive, emotional, and sociocultural factors (Zhang and Tian, 2024; Li, 2023). Beyond subjectivity, language acquisition is inherently multifaceted.

An effective strategy must address a range of elements: vocabulary development, grammatical understanding, speaking and listening fluency, cultural literacy, and learner motivation (Budiman et al., 2023; Newton and Nation, 2020). For example, a learner may excel at reading comprehension but struggle with spontaneous conversation due to insufficient exposure to authentic language use. Moreover, sustained motivation, especially in the face of the Japanese language's difficulty, plays a critical role in persistence and long-term success (Dörnyei and Henry, 2022).

Another complicating factor in evaluating Japanese language learning strategies is the considerable variability in instructional environments and methodologies. Educational institutions, private tutors, online platforms, and self-directed learners often adopt vastly different approaches ranging from immersive conversation-based techniques to grammar-translation methods and gamified mobile learning. Standardized tests like the Japanese Language Proficiency Test (JLPT) offer a useful benchmark but may not fully capture communicative competence or intercultural fluency.

Traditional evaluation models, which rely on fixed scoring systems, often fail to account for ambiguity and contextual variation in language use. Therefore, any effective assessment framework must be capable of handling subjectivity, imprecision, and multidimensional input. One promising solution is the application of Multi-Criteria Decision-Making (MCDM) methods, which provide a structured means of comparing diverse strategies across multiple evaluative dimensions. Incorporating fuzzy logic into these models further enhances their ability to accommodate uncertainty in expert judgment and learner experience, making evaluations more adaptive, accurate, and relevant to real-world language acquisition scenarios.

Previous studies have examined Japanese language learning from diverse perspectives, including grammar-based instruction, communicative language teaching, task-based learning, technology-assisted language learning, and cultural immersion (Richards and Rodgers, 2014; Long, 2014). Although these approaches have demonstrated positive effects on learner performance, most studies have evaluated instructional effectiveness using isolated indicators such as examination scores, learner satisfaction, or classroom observations. Consequently, the multidimensional nature of language learning has often been overlooked, limiting comprehensive comparisons among alternative instructional strategies (Japan, 2020).

Recent studies grounded in Second Language Acquisition theory have emphasized that successful language learning depends on the interaction among cognitive, motivational, instructional, and sociocultural factors rather than any single instructional component (Ellis, 2015). However, existing evaluation approaches rarely integrate these dimensions into a unified decision-making framework (Norris and Ortega, 2000; Chapelle, 2009). Furthermore, conventional statistical analyses generally assume precise numerical judgments and therefore have limited capacity to accommodate the uncertainty associated with expert opinions and learner perceptions. These limitations highlight the need for an evaluation framework that integrates multiple criteria and accounts for uncertainty in educational decision-making.

In recent years, Japanese language education has attracted growing attention, particularly amid global academic collaboration, cultural interest, and economic exchange. As institutions seek to enhance the quality and effectiveness of Japanese instruction, the need for a structured and comprehensive evaluation framework has become increasingly evident (Ekinci et al., 2022).

Traditional assessment approaches often fail to account for the multidimensional nature of language acquisition, which involves cognitive, affective, sociocultural, and pedagogical variables. Recognizing this complexity, recent research has proposed the use of Multi-Criteria Decision-Making (MCDM) methods, such as the MULTIMOORA approach under Plithogenic sets to address the ambiguity and uncertainty inherent in evaluating language teaching quality. By assigning weights to diverse criteria and ranking instructional alternatives, such models provide a mathematically grounded mechanism for navigating subjective judgments and diverse stakeholder priorities.

Building upon this foundation, the present study introduces a hybrid Fuzzy Analytic Hierarchy Process (FAHP) and Fuzzy Technique for Order of Preference by Similarity to Ideal Solution (FTOPSIS) framework for evaluating and ranking Japanese language learning strategies. FAHP is used to capture expert judgments regarding the relative importance of multiple criteria such as linguistic complexity, learner motivation, cultural immersion, technological integration, and learning flexibility, by translating qualitative comparisons into fuzzy weights. These weights reflect the degree of influence each criterion holds in determining the effectiveness of a learning strategy. Subsequently, FTOPSIS is employed to assess and rank specific language-learning methods by measuring their relative closeness to an ideal solution, thereby enabling a structured comparison of alternatives based on expert- and learner-informed input.

Although previous studies have investigated individual aspects of Japanese language learning, including learner motivation, instructional methods, technological support, and cultural immersion, relatively few studies have integrated these dimensions within a comprehensive evaluation framework. Existing research has also relied primarily on conventional statistical techniques that do not adequately represent the uncertainty inherent in expert judgments and learner evaluations. Moreover, direct comparisons among alternative Japanese language learning strategies remain limited. Therefore, a robust multi-criteria decision-making framework is required to provide a systematic and evidence-based evaluation of instructional effectiveness.

**Figure 1:**
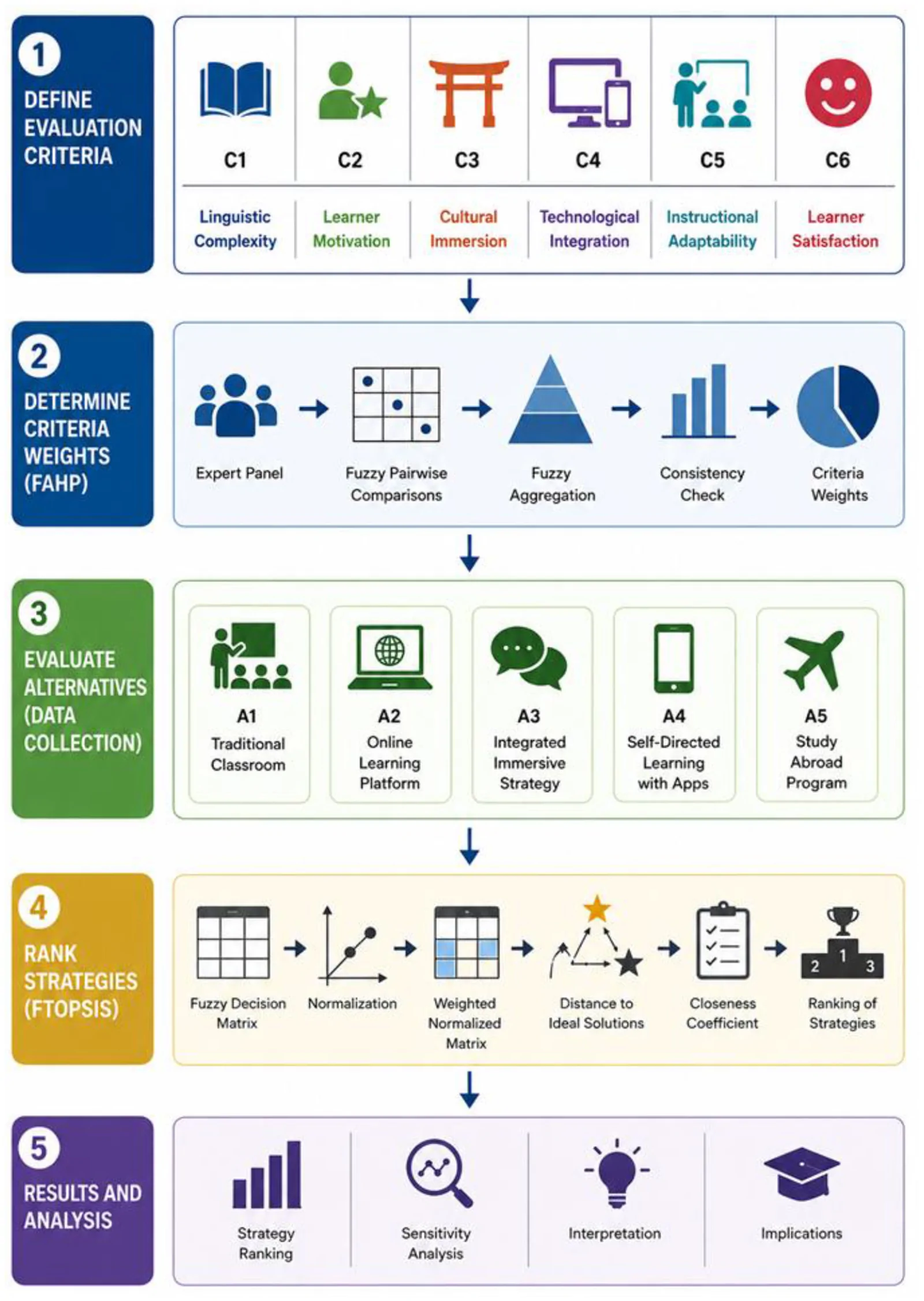
A structured flowchart representing the FAHP–FTOPSIS decision-making framework used to evaluate Japanese language learning strategies. The model incorporates expert judgments and literature-based insights to define criteria, calculate fuzzy weights, construct a decision matrix, and derive a ranked set of learning strategies based on their relative closeness to an ideal solution.

A visual representation of the evaluation process is provided in Figure 1. The structure of the paper is as follows: Section 2 outlines the methodology and data collection procedures; Section 3 details the implementation of the FAHP–FTOPSIS model; Section 4 presents the results and analysis; Section 5 discusses the implications; and Section 6 concludes with key findings and recommendations.

## 2 Methodology

The methodological framework adopted in this study is consistent with previous applications of FAHP and FTOPSIS in educational evaluation and multi-criteria decision making. FAHP has been widely used to determine criterion importance under uncertainty, whereas FTOPSIS has been used to rank alternatives by integrating multiple weighted criteria. Their combined application has been demonstrated to provide reliable and systematic decision support in complex educational environments (Kayashima et al., 2022; Goh et al., 2025; Islam, 2024; Dizon, 2021; Japan, 2020; Dörnyei, 2014; Kanno, 2008).

This study employed a hybrid Multi-Criteria Decision-Making framework integrating the Fuzzy Analytic Hierarchy Process (FAHP) and the Fuzzy Technique for Order of Preference by Similarity to Ideal Solution (FTOPSIS) to evaluate Japanese language learning strategies. To address these complexities, this study employs a hybrid Multi-Criteria Decision-Making (MCDM) framework that integrates the Fuzzy Analytic Hierarchy Process (FAHP) and the Fuzzy Technique for Order of Preference by Similarity to Ideal Solution (FTOPSIS).

The aim is to establish a structured, data-driven model to assess and rank Japanese language learning strategies by incorporating both expert judgments and learner-based evaluations. The methodological design follows a quantitative approach grounded in structured questionnaires administered to language learners and experts across diverse instructional settings, including universities, language centers, and online programs. FAHP is used to capture expert consensus on the relative importance of key evaluation criteria.

At the same time, FTOPSIS facilitates the ranking of learning strategies based on their weighted performance across these dimensions. The criteria were identified through a comprehensive literature review and expert consultation, ensuring both theoretical grounding and practical relevance. Specifically, the evaluation model considers six core dimensions of language learning effectiveness: linguistic complexity, learner motivation, cultural immersion, technological integration, instructional adaptability, and learner satisfaction. These dimensions are integrated into the FAHP-FTOPSIS framework to translate qualitative feedback into quantifiable outcomes.

The combined use of fuzzy logic and multi-criteria decision-making not only enhances the robustness of the evaluation process but also mitigates the uncertainty and subjectivity inherent in educational assessments. A visual overview of the evaluation criteria and their integration into the decision-making framework is provided in Figure 1.

The implementation of the hybrid FAHP–FTOPSIS framework followed eight sequential steps to ensure methodological transparency and reproducibility. First, the evaluation objective was defined as the assessment of Japanese language learning strategies. Second, six evaluation criteria were identified through a literature review and consultation with five experts in Japanese language education.

Third, the experts completed pairwise comparison matrices using linguistic judgments represented by triangular fuzzy numbers (TFNs). Fourth, the fuzzy comparison matrices were aggregated, fuzzy weights were calculated using the geometric mean method, defuzzified using the centroid method, normalized, and verified for consistency (CR < 0.10). Fifth, learner responses from 30 participants were collected through a structured questionnaire using a five-point Likert scale and converted into fuzzy linguistic values to construct the fuzzy decision matrix.

Sixth, the decision matrix was normalized and multiplied by the FAHP criterion weights. Seventh, the fuzzy positive ideal solution (FPIS) and fuzzy negative ideal solution (FNIS) were determined, Euclidean distances were calculated, and closeness coefficients were obtained for each learning strategy. Finally, the learning strategies were ranked by their closeness coefficients, and the stability of the ranking was assessed through sensitivity analysis with ±30% variations in the three highest-weighted criteria.

Table 1 summarizes the sequential implementation of the hybrid FAHP–FTOPSIS framework adopted in this study, providing a transparent and reproducible overview of each computational step.

**Table 1:** Step-by-step implementation of the FAHP–FTOPSIS framework.

Step	Procedure	Output
1	Define evaluation criteria	Criteria hierarchy
2	Expert pairwise comparison	Fuzzy comparison matrix
3	Calculate FAHP weights	Criterion weights
4	Consistency test	CR < 0.10
5	Collect learner responses	Decision matrix
6	Normalize matrix	Normalized matrix
7	Apply criterion weights	Weighted matrix
8	Determine FPIS and FNIS	Reference solutions
9	Calculate distances	D^+^, D^−^
10	Compute the closeness coefficient	Final ranking
11	Sensitivity analysis	Robustness verification

### 2.1 Data collection and questionnaire design

To evaluate Japanese language learning strategies, data were collected through a structured questionnaire administered to 30 Japanese language learners affiliated with Hunan University of Science and Technology. Participants were selected through purposive sampling based on their active engagement in Japanese-language study. To capture variation in learner characteristics, the sample included individuals of different ages and proficiency levels. Participants were categorized into three age groups: 18–22 years (*n* = 12), 23–27 years (*n* = 10), and 28–35 years (*n* = 8). In addition, learners were classified according to their Japanese language proficiency as beginner (*n* = 13), intermediate (*n* = 11), and advanced (*n* = 6), based on self-reported proficiency and institutional placement records.

To improve the transparency and reproducibility of the participant selection process, clear inclusion and exclusion criteria were established before data collection. Participants were required to be currently enrolled in a Japanese language course, actively engaged in Japanese language learning during the study period, at least 18 years of age, willing to provide informed consent, and to complete the entire questionnaire. Individuals who were not enrolled in Japanese-language study, had no recent learning experience, were younger than 18 years of age, declined to participate, or submitted incomplete questionnaires were excluded from the analysis. These criteria ensured that the study sample was appropriate for the research objectives and that the collected data were reliable for subsequent analysis.

The questionnaire was developed following a comprehensive review of the literature on Second Language Acquisition, Japanese language education, and educational evaluation, as well as consultation with five experts in Japanese language education. The six evaluation criteria, namely Linguistic Complexity, Learner Motivation, Cultural Immersion, Technological Integration, Instructional Adaptability, and Learner Satisfaction, were selected because they have consistently been identified as key determinants of language-learning effectiveness in previous studies (Japan, 2020; Ellis, 2015; Dörnyei, 2014).

**Figure 2:**
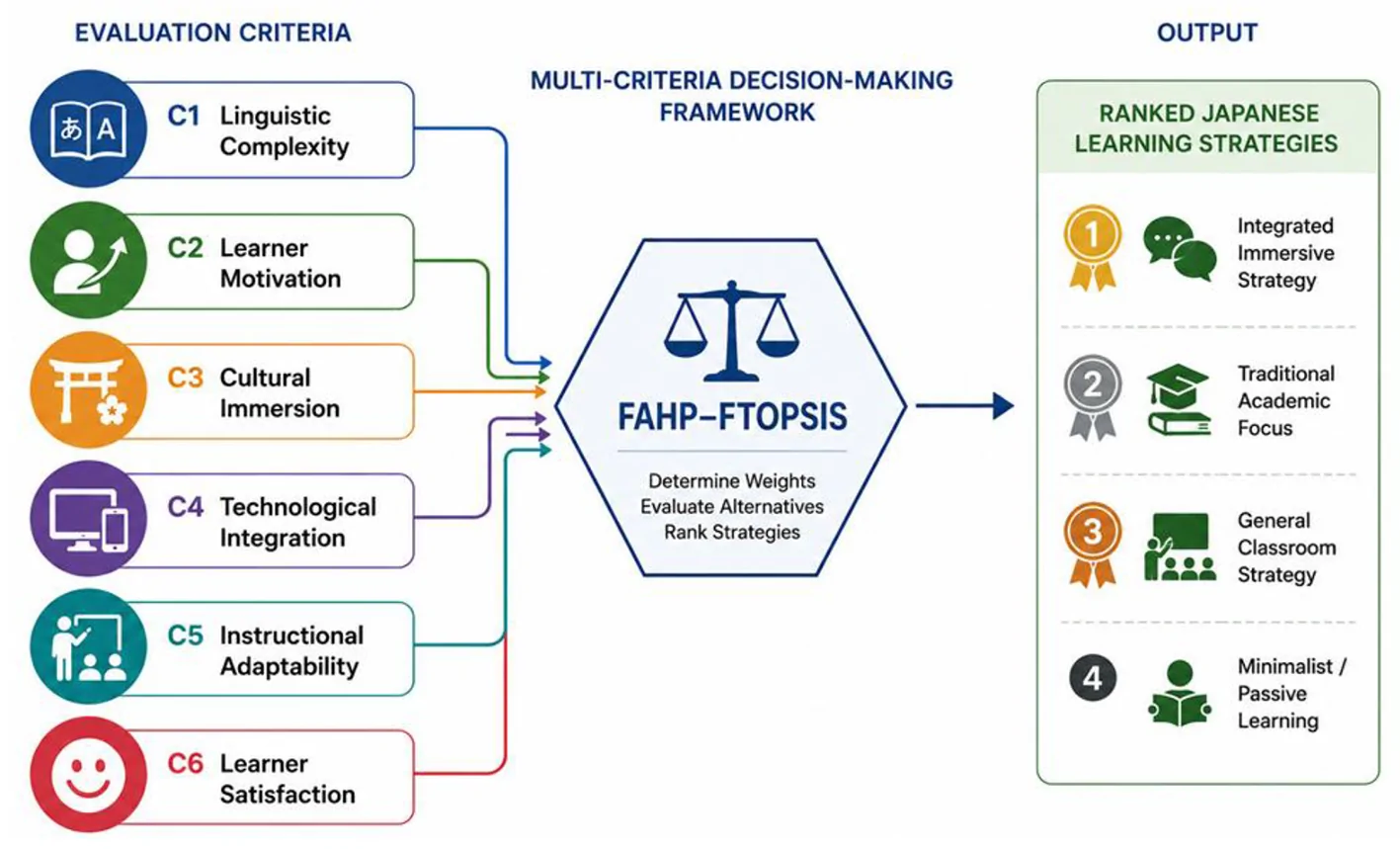
A conceptual framework for evaluating Japanese language learning strategies using the FAHP–FTOPSIS model. Six core criteria, linguistic complexity, learner motivation, cultural immersion, technological integration, instructional adaptability, and learner satisfaction, serve as multidimensional inputs into the evaluation model. The integrated FAHP–FTOPSIS process systematically analyses and ranks learning strategies based on their relative proximity to an ideal solution.

Six evaluation criteria were included: Linguistic Complexity, Learner Motivation, Cultural Immersion, Technological Integration, Instructional Adaptability, and Learner Satisfaction (Figure 2). All items were measured using a five-point Likert scale ranging from 1 (strongly disagree) to 5 (strongly agree). Prior to data collection, the questionnaire was reviewed by experts in Japanese language education to ensure content validity. The data collection process was further supported by a panel of Japanese-language educators who participated in identifying and weighting evaluation criteria for the FAHP analysis.

### 2.2 FAHP implementation

To determine the relative importance of the evaluation criteria, the Fuzzy Analytic Hierarchy Process (FAHP) was employed to analyze expert judgments and derive criterion weights under conditions of uncertainty. The FAHP process began with the construction of a hierarchical model composed of the overall goal (evaluating language learning strategy effectiveness), the six main criteria (Linguistic Complexity, Learner Motivation, Cultural Immersion, Technological Integration, Instructional Adaptability, and Learner Satisfaction), and the alternatives (learning strategies to be evaluated). The FAHP implementation consisted of five computational stages:

Step 1: Construct the fuzzy pairwise comparison matrix using expert judgments expressed as triangular fuzzy numbers.
Step 2: Aggregate individual expert matrices through the fuzzy geometric mean method.
Step 3: Calculate fuzzy synthetic weights for each criterion.
Step 4: Defuzzify the fuzzy weights using the centroid method to obtain crisp values, then normalize them so that their sum equals 1.
Step 5: Evaluate the consistency of expert judgments by calculating the consistency ratio (CR). When CR exceeded 0.10, the experts revised their pairwise comparisons until acceptable consistency was obtained.

A panel of five experts in Japanese language education and curriculum development participated in the pairwise comparison process. All experts had more than 5 years of experience in language teaching, curriculum design, or educational assessment. This step generated fuzzy weights that captured the range of expert judgments across all criteria. The fuzzy weights were defuzzified using the centroid method to facilitate quantitative analysis, producing crisp numerical weights. These normalized values represent the final priority of each criterion and serve as inputs for the subsequent FTOPSIS analysis. A consistency check was performed using the Consistency Ratio (CR) to assess the logical coherence of expert judgments. If the CR exceeded the acceptable threshold of 0.1, experts were asked to revise their comparisons. This quality control measure enhanced the validity and reliability of the weight estimation process.

### 2.3 FTOPSIS implementation

Following the derivation of criterion weights using FAHP, the Fuzzy Technique for Order of Preference by Similarity to Ideal Solution (FTOPSIS) was applied to evaluate and rank the effectiveness of different Japanese language learning strategies. FTOPSIS is a robust multi-criteria decision-making technique that compares each alternative based on its closeness to the ideal solution and its distance from the negative ideal solution. It is particularly well-suited for comparative educational assessments. The implementation of FTOPSIS followed six sequential stages.

Step 1: Construct the fuzzy decision matrix from learner questionnaire responses.
Step 2: Convert Likert scale responses into triangular fuzzy numbers.
Step 3: Normalize the fuzzy decision matrix.
Step 4: Multiply the normalized matrix by the FAHP criterion weights to obtain the weighted normalized matrix.
Step 5: Determine the fuzzy positive ideal solution (FPIS) and fuzzy negative ideal solution (FNIS), and calculate the Euclidean distance of each alternative from both reference solutions.
Step 6: Calculate the closeness coefficient using as Equation 1

$$\begin{array}{l} \text{C}{\text{C}}_{\text{i}}=\frac{{\text{D}}_{\text{i}}^{-}}{{\text{D}}_{\text{i}}^{+}+{\text{D}}_{\text{i}}^{-}} \end{array}$$ (1)

where $D_{i}^{+}$ is the distance to the FPIS and $D_{i}^{-}$ is the distance to the FNIS. The alternatives were ranked in descending order of the closeness coefficient.

### 2.4 Data aggregation

To ensure the reliability, transparency, and efficiency of the FAHP–FTOPSIS implementation, this study employed a combination of statistical and computational tools for data aggregation, fuzzy processing, and matrix analysis. Integrating expert evaluations and learner responses required both manual and automated procedures to transform qualitative judgments into quantifiable outputs. The data aggregation process began with collecting expert inputs through structured pairwise comparison matrices for the FAHP phase. These inputs were encoded using triangular fuzzy numbers to capture the inherent uncertainty in linguistic judgments (e.g., “moderately more important” or “strongly less important”).

A fuzzy pairwise comparison matrix was constructed, and the fuzzy geometric mean method was applied to compute preliminary fuzzy weights for each criterion. The weights were defuzzified using the centroid method to obtain crisp numerical values, which were subsequently normalized to ensure consistency and comparability across criteria. Simultaneously, learner evaluations were collected via a structured questionnaire administered to 30 participants enrolled in various Japanese language programs. The questionnaire, built around the six main evaluation criteria: Linguistic Complexity, Learner Motivation, Cultural Immersion, Technological Integration, Instructional Adaptability, and Learner Satisfaction, used a five-point Likert scale to capture perceptions of strategy effectiveness.

These responses were aggregated and averaged across participants to construct the decision matrix, with each row representing a learning strategy and each column corresponding to an evaluation criterion. The FAHP-derived weights were applied to the normalized decision matrix during the FTOPSIS phase. Each score was adjusted based on the importance of its corresponding criterion, yielding a weighted, normalized matrix. This matrix identified the Fuzzy Positive Ideal Solution (FPIS) and Fuzzy Negative Ideal Solution (FNIS) by selecting the best and worst scores for each criterion.

The distance of each strategy from these ideal and anti-ideal solutions was calculated using fuzzy Euclidean distance formulas, enabling the computation of the Closeness Coefficient (CC) for each alternative. All computations, including fuzzy arithmetic, defuzzification, normalization, and ranking, were implemented using Python (NumPy and Pandas for matrix operations and Scikit-Fuzzy for fuzzy logic handling).

To facilitate reproducibility, all preprocessing and computational procedures were performed in the same sequence for every experiment. Missing responses were checked before analysis; pairwise comparison matrices were verified for completeness; criterion weights were normalized prior to the FTOPSIS analysis; and the final ranking was independently verified using MATLAB. The complete workflow can therefore be replicated using the same questionnaire responses, expert comparison matrices, and computational sequence.

Microsoft Excel was used for data entry, initial sorting, and verification, while MATLAB was employed to cross-check calculations for accuracy. This hybrid toolchain ensured robustness, minimized computational errors, and facilitated reproducibility. The final output of the data aggregation and analysis process was a ranked list of Japanese language learning strategies; each associated with a closeness coefficient. These results formed the basis for the subsequent sensitivity analysis and final recommendations.

## 3 Results and discussion

Accurately evaluating the effectiveness of Japanese language learning strategies requires a structured, multi-criteria approach that captures both learners' nuanced experiences and the methodological precision of expert judgment. Unlike single-metric evaluations, language acquisition involves a wide array of interdependent factors, including linguistic complexity, cultural context, learner motivation, instructional adaptability, technological integration, and satisfaction.

While often difficult to quantify, these dimensions significantly influence how different learning strategies perform in diverse instructional settings. Traditional evaluation methods fail to address these complexities, usually relying on isolated feedback or narrowly scoped performance metrics. This study applied an integrated FAHP–FTOPSIS framework to overcome these limitations by combining fuzzy multi-criteria decision-making with empirical data from learners and experts. This hybrid approach enables the systematic weighting of criteria using expert knowledge (via FAHP) and the objective ranking of alternatives using learner-reported outcomes (via FTOPSIS).

This framework assessed four distinct Japanese language learning strategies across six evaluation criteria, providing a comprehensive, data-driven basis for identifying the most effective pedagogical models. The results reveal how each strategy aligns with ideal learning outcomes and underscore the value of fuzzy logic in handling subjectivity, ambiguity, and multidimensionality in educational assessments.

### 3.1 Descriptive statistical analysis

To capture overall response trends and assess the internal consistency of participant feedback, this study employed key descriptive statistical measures, namely, arithmetic mean, variance, and standard deviation, for each item included in the evaluation questionnaire. These metrics serve as foundational tools in Likert-scale research, enabling the interpretation of central tendencies and the dispersion of responses across participants. Descriptive statistics were calculated to summarize learner responses across the six evaluation criteria.

Mean scores, variances, and standard deviations were used to examine overall response patterns and the consistency of participant perceptions regarding Japanese language learning strategies. These statistical procedures were applied to all 20 questionnaire items, which were distributed across six primary evaluation criteria. Responses were collected from 30 participants, ensuring a diverse yet focused dataset for analysis. The results revealed consistently high mean scores across most items, with most exceeding 4.0 on a five-point Likert scale. This indicates that learners generally held favorable perceptions of the Japanese-language learning strategies they encountered.

The standard deviation values, ranging from 0.9 to 1.1, suggest moderate response variability. While individual experiences varied, the overall pattern reflects high internal consistency and reliability in participant feedback. This balance between central tendency and dispersion reinforces the credibility of the collected data and supports its use in subsequent FAHP–FTOPSIS analysis. A summary of descriptive statistics for representative items under each evaluation criterion is presented in Table 2.

**Table 2:** Descriptive statistics of selected questionnaire items.

Evaluation criterion	Questionnaire item (summary)	Mean	Variance	Std. deviation
Linguistic complexity	The program improved my grammar and vocabulary understanding	4.05	1.07	1.04
Learner motivation	I remained motivated throughout the learning process	4.18	0.81	0.90
Cultural immersion	The course offered meaningful exposure to Japanese culture	4.03	0.89	0.94
Technological integration	The digital tools used enhanced my learning	4.15	0.88	0.94
Instructional adaptability	Teaching methods adapted to my learning needs	4.08	0.93	0.96
Learner satisfaction	I am satisfied with the overall learning experience	4.10	0.95	0.97

Each evaluation criterion represents a distinct dimension of Japanese language learning effectiveness. Linguistic Complexity measures the development of grammar, vocabulary, and language structures. Learner Motivation reflects sustained engagement and willingness to continue learning. Cultural Immersion evaluates learners' exposure to authentic Japanese language and culture. Technological Integration measures the contribution of digital learning resources to language acquisition. Instructional Adaptability reflects the ability of teaching approaches to accommodate different learner needs, while Learner Satisfaction represents the overall perceived quality and effectiveness of the learning experience. Together, these dimensions provide a comprehensive evaluation of instructional effectiveness.

Table 2 presents the descriptive statistics for selected items corresponding to the six evaluation criteria used to assess the effectiveness of Japanese language learning strategies. These metrics provide critical insight into learner perceptions and the degree of consistency across responses, forming an empirical foundation for the subsequent FAHP–FTOPSIS analysis. Based on a five-point Likert scale (1 = Strongly Disagree, 5 = Strongly Agree), the mean values indicate that all criteria received average scores above 4.0. This suggests a broadly positive assessment of the learning strategies implemented.

Among the six dimensions, Learner Motivation achieved the highest mean score (4.18), reflecting sustained student engagement and emotional investment in learning. Technological Integration (4.15) and Instructional Adaptability (4.08) followed closely, underscoring the perceived utility of digital tools and the responsiveness of instructional methods to individual learning needs. Learner Satisfaction (4.10) and Linguistic Complexity (4.05) also received favorable mean scores, indicating satisfaction with program quality and with the effectiveness of language-structure instruction. Although Cultural Immersion recorded the lowest mean (4.03), the score still reflected an overall positive evaluation, suggesting some variability in the depth or authenticity of cultural experiences across different strategies.

Analysis of variance and standard deviation values offers further insight into response consistency. Learner Motivation showed the lowest variability (variance: 0.81, SD: 0.90), indicating a strong consensus regarding the programs' motivational impact. In contrast, Linguistic Complexity exhibited the highest variance (1.07) and standard deviation (1.04), indicating greater variation in learner experiences, potentially due to differences in prior knowledge, instructional clarity, or the linguistic demands of specific curricula.

**Figure 3:**
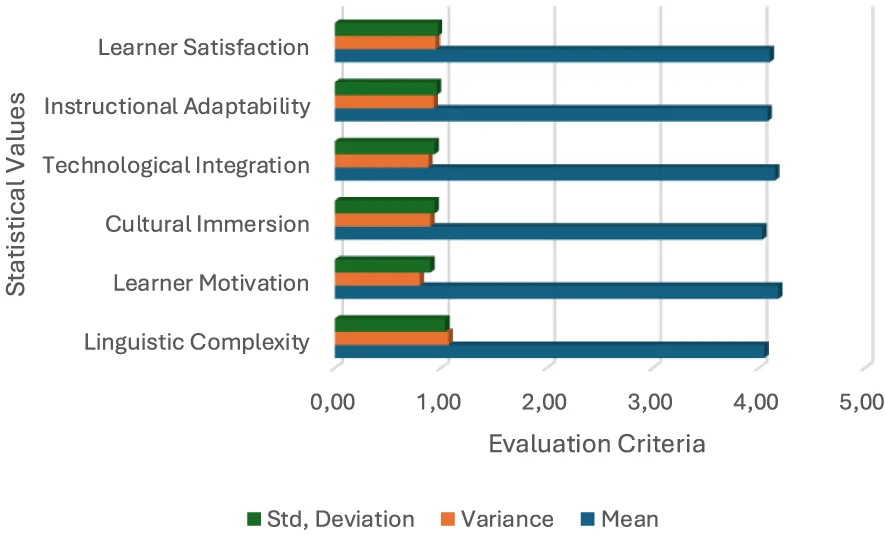
Comparison of descriptive statistical measures—mean, variance, and standard deviation, for six evaluation criteria related to Japanese language learning strategies. The chart demonstrates generally high mean scores across all criteria, with learner motivation showing the highest consistency and linguistic complexity reflecting the greatest variability.

Other criteria, including Cultural Immersion, Technological Integration, and Learner Satisfaction, demonstrated moderate variability, with standard deviations ranging from 0.94 to 0.97. Instructional Adaptability presented a standard deviation of 0.96, suggesting relatively consistent but slightly more varied experiences depending on the degree of personalization offered. The findings indicate that all six dimensions were positively evaluated, with Motivation, Technology, and Instructional Adaptability emerging as relative strengths. Meanwhile, the greater variation in responses related to linguistic complexity may signal opportunities to refine curriculum design to better match learners' needs and proficiencies. The distributions of mean, variance, and standard deviation across all six evaluation criteria are illustrated in Figure 3, highlighting both central tendency and variability in learner responses.

### 3.2 FAHP weight calculation

FAHP analysis was conducted to determine the relative importance of the six evaluation criteria. The results indicated that Linguistic Complexity received the highest weight (22.1%), followed by Cultural Immersion (20.4%) and Learner Motivation (17.8%). Instructional Adaptability and Learner Satisfaction received the lowest weights. These findings suggest that experts place greater emphasis on linguistic mastery and authentic cultural engagement when evaluating Japanese language learning strategies.

The prioritization of Linguistic Complexity and Cultural Immersion is consistent with Second Language Acquisition research, which argues that grammatical competence and authentic cultural exposure jointly support communicative competence and long-term language development (Ellis, 2004, 2015). Similarly, Dörnyei (2014) emphasized that learner motivation complements, rather than replaces, linguistic development, a point reflected in the third-highest criterion weight assigned by the expert panel (Dörnyei, 2014).

**Figure 4:**
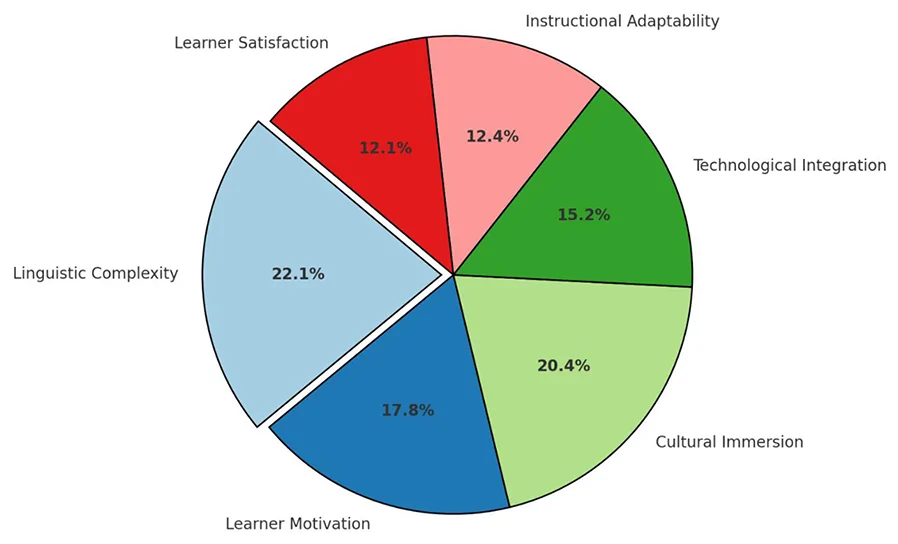
FAHP-derived weight distribution across the six evaluation criteria for Japanese language learning strategies. Linguistic Complexity holds the largest share (22.1%), followed by cultural immersion and learner motivation, indicating that experts place the greatest emphasis on structural language mastery and immersive learning experiences.

Experts are allowed to express pairwise comparisons using linguistic scales (e.g., “equally important,” “strongly more important”), which are then translated into triangular fuzzy numbers (TFNs). These fuzzy inputs form the basis of a pairwise comparison matrix, from which fuzzy geometric means are computed to generate preliminary weight estimates. Subsequently, the fuzzy weights were defuzzified using the centroid method to facilitate interpretability and application during the FTOPSIS phase, yielding crisp numerical weights that represent each criterion's relative importance (see Figure 4).

To empirically validate the FAHP-derived weights, this section compares the importance of expert-assigned criteria with learner-reported perceptions across distinct demographic segments, specifically, age and proficiency levels. This approach helps ensure expert judgments reflect actual learner experiences and provides insight into how learning priorities evolve across user profiles. Table 3 presents the mean scores for each evaluation criterion across age groups: 18–22, 23–27, and 28–35 years. The results indicate a clear developmental trend in strategic preferences.

**Table 3:** Mean scores by age group across evaluation criteria.

Evaluation criterion	18–22 years	23–27 years	28–35 years	Observations
Linguistic complexity	4.02	4.12	4.25	Highest in the oldest group; advanced learners prioritize grammar/vocab mastery.
Learner motivation	4.20	4.18	4.14	There is a slight decline with age; motivation remains high across groups.
Cultural immersion	4.00	4.10	4.20	Older learners seek a deeper cultural context.
Technological integration	4.22	4.15	4.10	Highest in the youngest group; tech-savvy preferences.
Instructional adaptability	4.10	4.08	4.06	Minor decrease with age; still consistently valued.
Learner satisfaction	4.18	4.12	4.05	Declines slightly with age; early-stage learners are more easily satisfied.

Younger learners (18–22) placed the highest emphasis on Technological Integration (mean = 4.22) and Learner Satisfaction (mean = 4.18), suggesting a preference for engaging, user-friendly digital environments and positive affective experiences. This aligns with generational familiarity with digital learning tools and a desire for immediate feedback and motivation. In contrast, older learners (28–35) rated Linguistic Complexity (mean = 4.25) and Cultural Immersion (mean = 4.20) more highly, indicating a shift in focus toward depth of language structure and real-world applicability.

These preferences closely mirror the FAHP-derived weights, prioritizing Linguistic Complexity (22.1%) and Cultural Immersion (second highest). The trend suggests that experienced learners increasingly value nuanced grammar and authentic language use—core elements of long-term proficiency.

Table 4 explores learner perceptions across proficiency levels (Beginner, Intermediate, and Advanced). As expected, Beginner learners prioritized Instructional Adaptability and Technological Integration, reflecting their need for support and flexible resources in the early learning stages. Advanced learners, on the other hand, reported the highest scores for Linguistic Complexity (mean = 4.30) and Cultural Immersion (mean = 4.18), reaffirming expert emphasis on these criteria for deeper linguistic mastery. Interestingly, Learner Satisfaction showed a modest decline from beginner to advanced levels, possibly reflecting a growing critical awareness of the effectiveness of strategies as learners progress.

**Table 4:** Mean scores by proficiency level across evaluation criteria.

Evaluation criterion	Beginner	Intermediate	Advanced	Observations
Linguistic complexity	4.00	4.10	4.30	Rises with proficiency; core focus at advanced stages.
Learner motivation	4.20	4.15	4.10	There was a slight decrease; it was still relatively high.
Cultural immersion	4.05	4.10	4.18	Advanced learners value cultural context more.
Technological integration	4.18	4.14	4.08	It is more useful for beginners seeking intuitive tools.
Instructional adaptability	4.12	4.08	4.05	Stable across levels; slightly higher in beginners.
Learner satisfaction	4.20	4.10	4.05	It decreases as learners develop critical evaluation skills.

**Figure 5:**
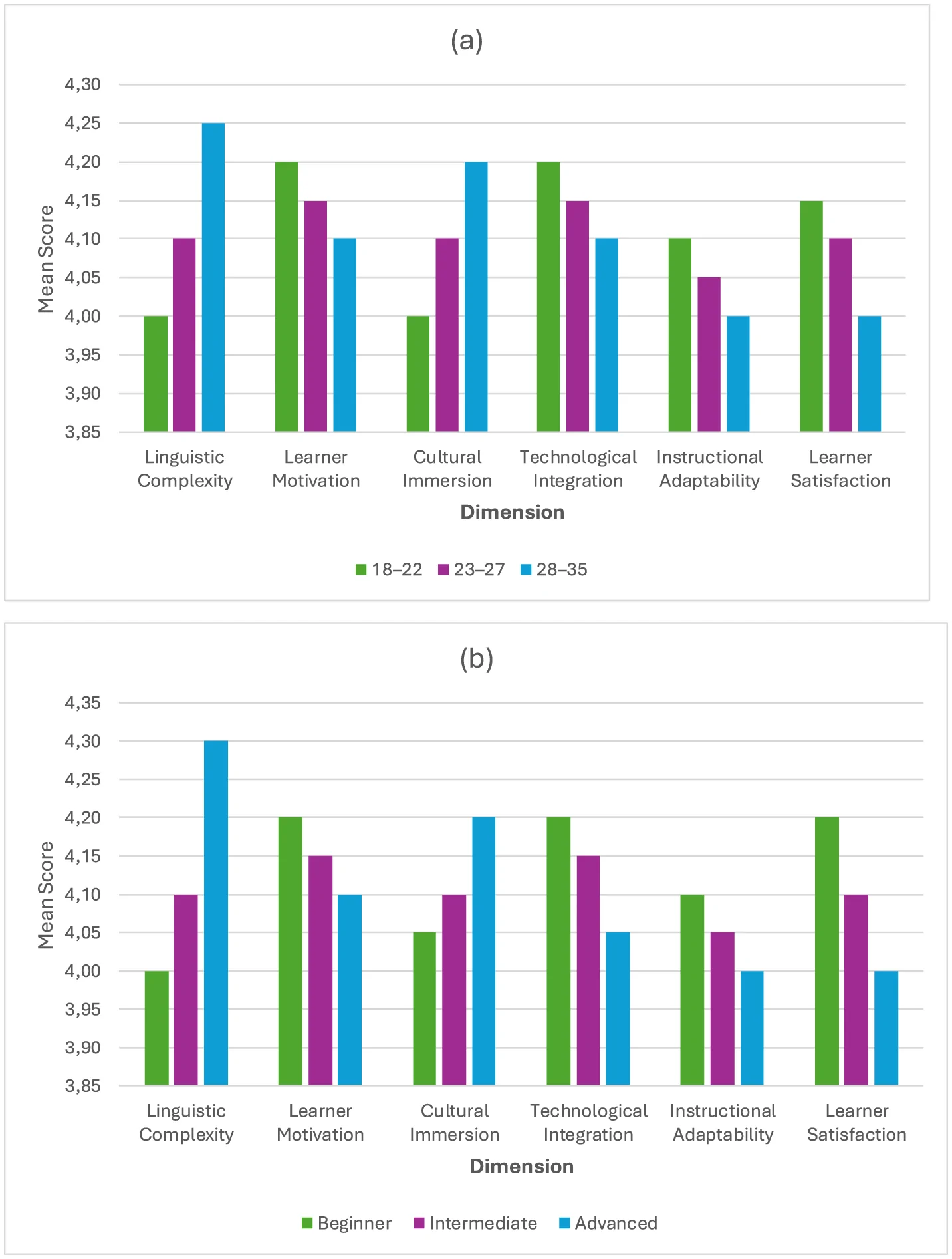
Comparative analysis of learner-reported mean scores across evaluation criteria for Japanese language learning strategies. **(A)** Responses segmented by age group (18–22, 23–27, and 28–35 years) show an increasing emphasis on linguistic complexity and cultural immersion with age, while younger learners prioritize technological integration and learner satisfaction. **(B)** Responses segmented by proficiency level (beginner, intermediate, advanced) reveal a similar trend: advanced learners value linguistic complexity and cultural immersion more highly, whereas beginners emphasize adaptability and motivational aspects. The grayscale palette and external legends enhance clarity and professional presentation.

These comparisons highlight a strong alignment between expert priorities and learner perceptions, especially in the later stages of language acquisition. While all criteria received positive evaluations, the increasing importance of linguistic rigor and immersion with age and proficiency supports the FAHP model's focus on foundational and experiential components (Figure 5).

### 3.3 FTOPSIS ranking of Japanese language learning strategies

The Fuzzy Technique for Order of Preference by Similarity to Ideal Solution (FTOPSIS) was employed to evaluate and rank Japanese language learning strategies based on their effectiveness. This method compares alternatives, in this case, specific learning strategies, by calculating their relative proximity to an ideal learning profile, using learner evaluations and FAHP-derived criterion weights. The normalized decision matrix was built from the aggregated mean scores reported by participants for each of the six criteria: Linguistic Complexity (22.1%), Learner Motivation (17.8%), Cultural Immersion (20.4%), Technological Integration (15.2%), Instructional Adaptability (12.4%), and Learner Satisfaction (12.1%). To facilitate practical evaluation, participant feedback was synthesized into four representative strategy models:

#### 3.3.1 Program A: integrated immersive strategy

A comprehensive, learner-centered approach that combines structured grammar instruction, cultural immersion (e.g., media, real-life exposure), personalized feedback, and interactive technology. High learner satisfaction and motivation, along with strong performance across all criteria, make this the benchmark model.

These findings are consistent with previous studies demonstrating that communicative and immersive instructional approaches promote stronger language acquisition than isolated grammar-based instruction (Ellis, 2015). The integration of technological resources with authentic cultural experiences also supports learner autonomy and sustained motivation, both of which have been identified as important predictors of successful second-language acquisition (Dörnyei, 2014). Therefore, the superior performance of the Integrated Immersive Strategy is supported by both the empirical findings of this study and established SLA theory.

#### 3.3.2 Program B: traditional academic focus

This is a curriculum-heavy, exam-oriented method emphasizing kanji mastery, grammar drills, and vocabulary acquisition. While strong in linguistic complexity and motivation (for goal-driven learners), it lacks adaptive teaching and cultural engagement and uses limited technology.

#### 3.3.3 Program C: general classroom strategy

A standard, mixed-method classroom approach. Moderate across the board: offers some structure and engagement but lacks innovation, individualized instruction, or immersive content. Student satisfaction varies widely.

#### 3.3.4 Program D: minimalist/passive learning

It relies heavily on rote memorization or low-interaction platforms with minimal immersion and adaptability. It often lacks motivation-building elements and authentic exposure to Japanese culture. It is the lowest rated across most dimensions.

The four learning strategies defined above provide a practical and conceptually rich representation of varying levels of effectiveness grounded directly in learner feedback. This structure enabled the meaningful application of the FTOPSIS method to objectively rank the strategies. An ideal solution representing the best possible weighted performance across all evaluation criteria, and a negative-ideal solution representing the lowest acceptable performance, were first established to assess overall performance. Each program's proximity to these benchmarks was evaluated using Euclidean distance calculations in a fuzzy multi-criteria space. These distances were then used to compute a similarity index for each strategy using the formula as Equation 2:


$$\begin{array}{l} S_{i}=\frac{d_{i}^{-}}{d_{i}^{+}+d_{i}^{-}} \end{array}$$ (2)

where $d_{i}^{+}$ is the distance to the ideal solution and $d_{i}^{-}$ is the distance to the negative-ideal solution. Programs with higher similarity scores are considered more effective because they are closer to the ideal profile for Japanese language learning (see Table 5). The FTOPSIS results below illustrate each strategy's performance based on its calculated distances from the ideal and non-ideal solutions.

**Table 5:** FTOPSIS results and ranking of Japanese language learning strategies.

language learning strategy	Distance to ideal solution (${\text{d}}_{\text{i}}^{+}$)	Distance to non-ideal solution (${\text{d}}_{\text{i}}^{-}$)	Similarity score (S_i_)	Rank
Program A	0.078	0.402	0.837	1
Program B	0.142	0.325	0.696	2
Program C	0.201	0.271	0.574	3
Program D	0.285	0.183	0.391	4

As Table 5 clearly shows, Program A (Integrated Immersive Strategy) achieved the highest closeness coefficient (0.837), indicating the strongest overall performance across the six evaluation criteria. This result suggests that integrating structured language instruction with cultural immersion, interactive technologies, and personalized learning support provides the most effective approach for Japanese language acquisition. The high ranking reflects balanced performance in linguistic development, learner motivation, and cultural competence, which were identified as the most influential evaluation criteria through the FAHP analysis.

Program B (Traditional Academic Focus) ranked second with a closeness coefficient of 0.696. Although this strategy performed well in linguistic complexity through intensive grammar and vocabulary instruction, its limited emphasis on cultural immersion, technological integration, and instructional flexibility reduced its overall effectiveness.

Program C (General Classroom Strategy) achieved a moderate closeness coefficient of 0.574. Its balanced but average performance across all evaluation criteria indicates that conventional classroom instruction provides acceptable learning outcomes but lacks the immersive and personalized elements required to maximize learner achievement.

Program D (Minimalist or Passive Learning) obtained the lowest closeness coefficient (0.391). The low ranking reflects weak performance across most evaluation dimensions, particularly learner motivation, instructional adaptability, and cultural immersion. These findings suggest that passive learning approaches that rely primarily on memorization are less effective at developing communicative competence and long-term engagement in Japanese language learning.

**Figure 6:**
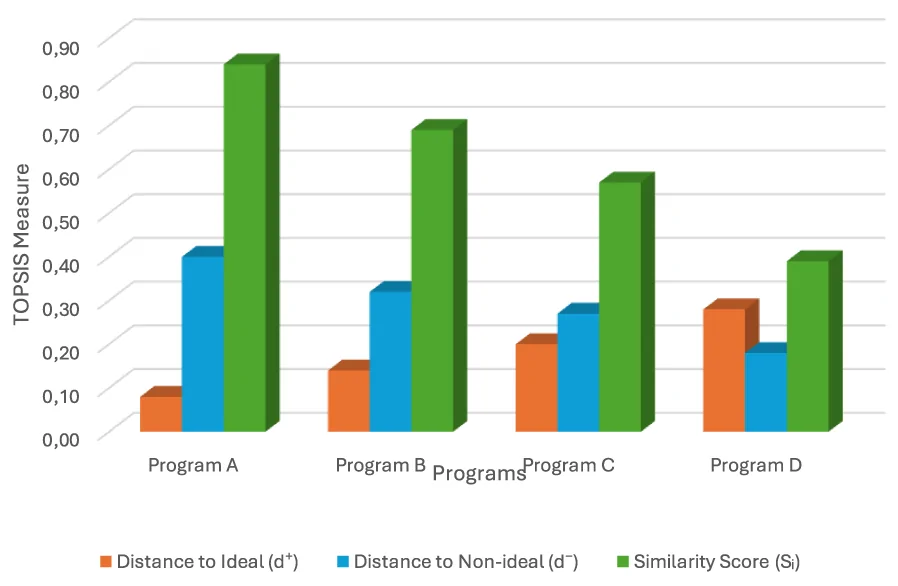
FTOPSIS-based comparison of four Japanese language learning strategies. The chart displays each program's distance to the ideal solution (d^+^), distance from the non-ideal solution (d^−^), and resulting similarity score (S_i_). Program A demonstrates the highest effectiveness with the smallest d^+^ and highest S_i_. At the same time, Program D ranks lowest across all performance metrics, indicating substantial gaps in instructional quality and learner alignment.

Figure 6 visually summarizes the evaluation results from Table 5, clearly illustrating the comparative performance of the four identified programs. Program A has the smallest distance to the ideal solution ($d_{i}^{+}$) and the greatest distance from the non-ideal solution ($d_{i}^{-}$), resulting in the highest similarity score (Si). Conversely, Program D shows the largest distance from the ideal and the lowest similarity score, indicating significant room for improvement. This graphical comparison distinctly highlights the relative effectiveness of each program, confirming Program A's superior performance across all evaluation criteria.

### 3.4 Sensitivity analysis

A sensitivity analysis was conducted to verify the robustness and reliability of the FAHP–FTOPSIS evaluation framework adopted in this study. This analysis systematically examined how changes in the relative importance (weights) of key evaluation criteria would affect the final rankings and similarity scores of the four Japanese language learning strategies (Programs A, B, C, and D).

Specifically, the model's sensitivity was tested against ±30% variations in three primary criteria: Linguistic Complexity, Cultural Immersion, and Learner Motivation. For each scenario, the selected criterion's weight was adjusted by ±30%, while the remaining weights were proportionally redistributed among the other five criteria to maintain a constant total of 100%. The resulting similarity scores were recalculated using the FTOPSIS method, and the rankings were compared with the baseline.

In the baseline scenario, the FAHP-derived weights were Linguistic Complexity (22.1%), Cultural Immersion (20.4%), Learner Motivation (17.8%), Technological Integration (15.2%), Instructional Adaptability (12.4%), and Learner Satisfaction (12.1%). The corresponding FTOPSIS similarity scores for Programs A, B, C, and D were 0.837, 0.696, 0.574, and 0.391, respectively.

**Figure 7:**
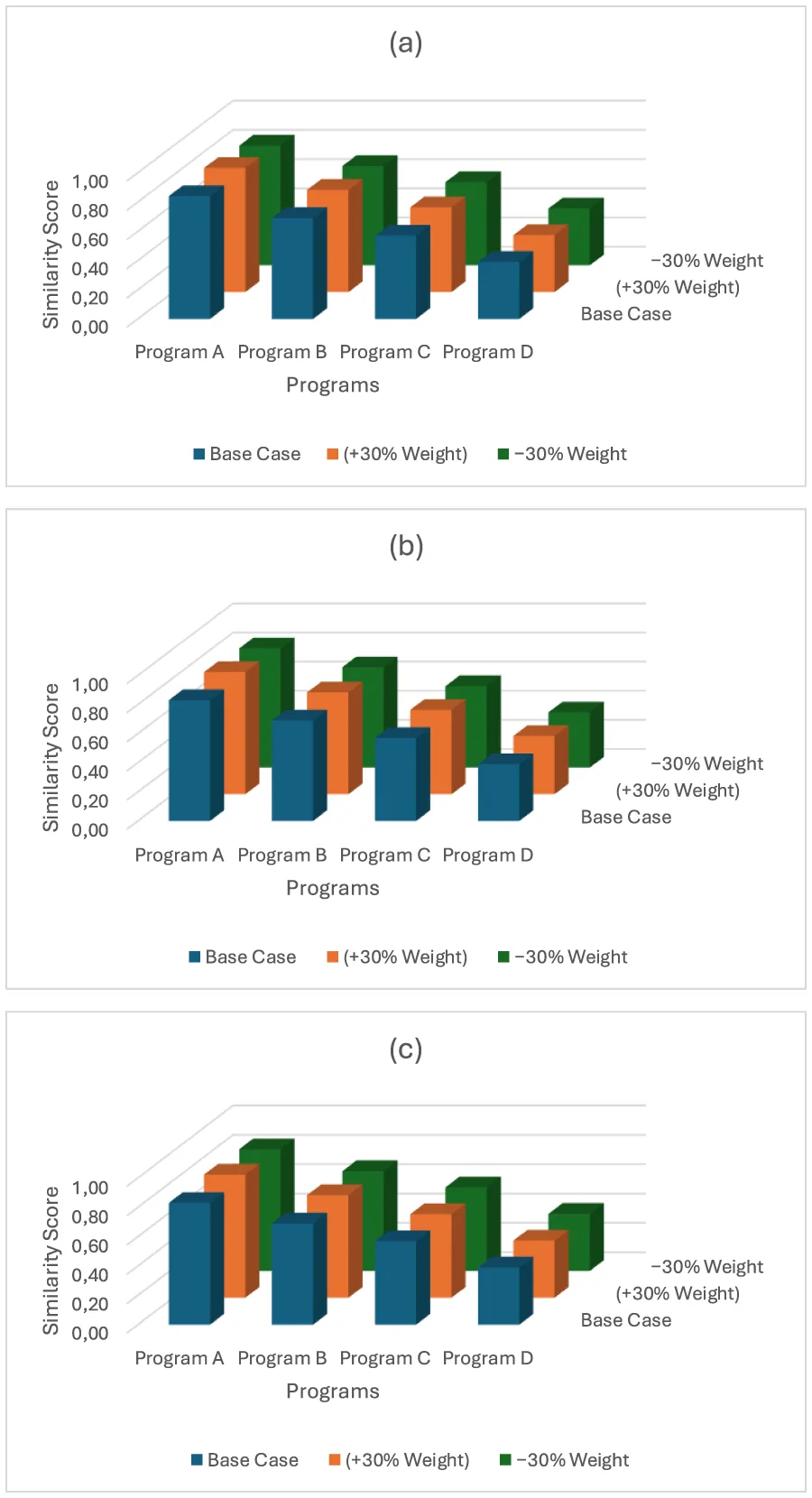
Sensitivity analysis illustrating the robustness of similarity scores for the evaluated Japanese language learning strategies (Programs A, B, C, D) to ±30% adjustments in evaluation criteria weights. **(A)** Linguistic complexity, **(B)** cultural immersion, and **(C)** learner motivation. Across all three scenarios, the relative rankings of the learning strategies remain stable, demonstrating the reliability and consistency of the FAHP–FTOPSIS evaluation framework employed in this study.

First, the model's sensitivity to changes in Linguistic Complexity was evaluated. Increasing this criterion's weight by 30% slightly improved the similarity scores for Programs A and B, reflecting their strong performance in structural language mastery. Conversely, decreasing its weight marginally lowered their scores. However, these changes did not affect the overall rankings, confirming model stability (Figure 7A). Second, the importance of cultural immersion varied by ±30%.

Programs A and D showed minor improvements when the weight increased, particularly Program A, which integrates immersive media and cultural exposure. When the weight was reduced, the similarity scores of Programs A and C declined slightly. Despite these fluctuations, the ranking order remained unchanged, reaffirming the model's consistency (Figure 7B). Lastly, the analysis focused on Learner Motivation, a critical affective component. Increasing its weight modestly boosted similarity scores for Programs A and B, which had high motivational engagement.

A 30% decrease in this weight slightly reduced their scores. Again, no change in relative rankings was observed (Figure 7C). The FAHP–FTOPSIS model produced consistent and stable rankings across all three scenarios. Although similarity scores were marginally affected by substantial shifts in criterion weights, the rank order of programs remained intact. These findings confirm the robustness and reliability of the proposed evaluation framework, highlighting its capacity to support informed decision-making in diverse and uncertain educational contexts.

The proposed methodology is fully reproducible because every stage of the FAHP and FTOPSIS implementation follows a predefined computational sequence, beginning with expert pairwise comparisons and ending with sensitivity analysis. The use of standard triangular fuzzy numbers, centroid defuzzification, normalized weights, Euclidean distance calculations, and clearly defined ranking procedures enables future researchers to replicate the evaluation using different learner populations or educational settings.

#### 3.4.1 Comparison with a baseline method

To further evaluate the proposed framework, the hybrid FAHP–FTOPSIS method was conceptually compared with the conventional TOPSIS approach, which was selected as the baseline because it is one of the most widely used multi-criteria decision-making techniques for ranking alternatives. Unlike the proposed framework, the conventional TOPSIS method assumes equal importance for all evaluation criteria unless predetermined weights are available and does not explicitly model the uncertainty associated with expert judgments. Consequently, the baseline method relies on crisp values throughout the decision-making process, whereas the proposed framework incorporates fuzzy logic to better represent the ambiguity inherent in educational evaluation.

The principal methodological differences between the two approaches are summarized in Table 6. The conventional TOPSIS model directly ranks alternatives based on normalized decision matrices and fixed criterion weights. In contrast, the proposed FAHP–FTOPSIS framework first derives criterion weights using the Fuzzy Analytic Hierarchy Process, based on expert pairwise comparisons expressed as triangular fuzzy numbers. These weights are then integrated into the FTOPSIS procedure to evaluate the relative performance of the learning strategies. This two-stage process enables the framework to capture both expert knowledge and learner perceptions while reducing the influence of subjective uncertainty.

**Table 6:** Comparison between the conventional TOPSIS method and the proposed FAHP–FTOPSIS framework.

Feature	Conventional TOPSIS	Proposed FAHP–FTOPSIS
Criterion weighting	Equal or predefined crisp weights	FAHP derived fuzzy weights from expert judgments
Treatment of uncertainty	Not considered	Represented using triangular fuzzy numbers
Expert knowledge	Limited	Incorporated through pairwise comparisons
Learner evaluations	Crisp values	Fuzzy linguistic assessments
Decision robustness	Moderate	Improved through fuzzy modeling and sensitivity analysis
Suitability for educational evaluation	Moderate	High

The comparison demonstrates several methodological advantages of the proposed framework. First, FAHP provides a systematic mechanism for determining the relative importance of evaluation criteria rather than assuming equal contributions from all factors. This distinction is particularly important in Japanese language learning, where linguistic complexity, cultural immersion, learner motivation, technological integration, instructional adaptability, and learner satisfaction do not contribute equally to learning effectiveness. Second, the incorporation of fuzzy logic allows expert opinions and learner responses to be represented more realistically by accommodating uncertainty and imprecision in linguistic judgments. Finally, the robustness of the proposed framework was further supported by the sensitivity analysis presented in Section 3.4, which demonstrated stable ranking results under substantial variations in criterion weights.

### 3.5 Discussions and limitations

This study employed a hybrid FAHP–FTOPSIS model to evaluate Japanese language learning strategies through a structured, multi-criteria lens. The results highlight significant distinctions in strategy effectiveness based on learner feedback and expert weighting of key educational criteria. The Integrated Immersive Strategy (Program A) consistently ranked among the evaluated alternatives, indicating a strong alignment between immersive, adaptable, and technology-enhanced learning environments and learner success. This affirms existing research emphasizing the importance of contextual engagement, learner autonomy, and multimodal inputs in second language acquisition.

The analysis also underscored the evolving nature of learner priorities across age and proficiency levels. Younger learners favored strategies that integrated technological tools and prioritized satisfaction, likely reflecting generational preferences for interactive and user-centered learning. In contrast, more advanced and older learners emphasized linguistic complexity and cultural immersion, suggesting a shift toward depth, authenticity, and long-term skill development. This trend corroborates the FAHP-generated criterion weights, affirming the model's validity. Furthermore, the consistency of the strategy rankings across sensitivity-analysis scenarios supports the robustness of the FAHP–FTOPSIS approach to managing ambiguity and subjectivity in educational assessments. The model successfully balanced qualitative learner experiences with quantitative expert input, offering a flexible framework that could be adapted to other language-learning contexts or to broader educational program evaluations.

Despite the comprehensive and structured nature of the FAHP–FTOPSIS framework, this study is subject to several limitations that may influence the generalizability and applicability of its findings. First, the sample size of 30 learners, though diverse in age and proficiency, was relatively small and geographically concentrated. This limited scope may not fully capture the broader variability in learner experiences across different cultural, institutional, or national contexts.

Additionally, the reliance on self-reported data introduces the possibility of response bias. Learner evaluations were based on subjective perceptions using Likert-scale items, which, while useful for capturing experiential insights, are susceptible to overestimation, social desirability effects, or inconsistent interpretations of scale values. Furthermore, the expert input used to weight the evaluation criteria in the FAHP phase, though guided by fuzzy logic to manage uncertainty, was still based on a relatively small panel of five specialists. Their judgments may reflect individual biases or disciplinary leanings, and a larger, more diverse expert panel could enhance the objectivity of the weightings.

The study examined only four representative learning strategies synthesized from commonly used pedagogical models. While these strategies were carefully constructed, they may not encompass the full range of existing or emerging approaches in Japanese language education, especially hybrid methods or those adapted to niche learner populations. The study's cross-sectional design also poses a limitation, as it captures learner feedback at a single point in time. Longitudinal research would be better suited to assess how learning outcomes and perceptions evolve, particularly about motivation, proficiency development, and engagement with cultural content.

Finally, the effectiveness of certain strategies, particularly those emphasizing immersive and technology-enhanced methods, may depend heavily on learners' access to resources such as native-speaker interaction, high-speed internet, and culturally authentic materials. Learners in resource-constrained settings may not experience these strategies, similarly, potentially limiting the scalability of the study's conclusions.

## 4 Conclusion

Evaluating language learning strategies requires integrating objective criteria and subjective learner experiences. The application of the FAHP–FTOPSIS model achieved this by assigning quantified importance to six key criteria, Linguistic Complexity (22.1%), Cultural Immersion (20.4%), Learner Motivation (17.8%), Technological Integration (15.2%), Instructional Adaptability (12.4%), and Learner Satisfaction (12.1%), and using these to rank four distinct Japanese learning strategies. Among these, the Integrated Immersive Strategy (Program A) emerged as the most effective, with a similarity score of 0.837, indicating its strong alignment with expert-defined benchmarks and learner expectations.

Notably, the analysis revealed clear patterns in learner preferences across age and proficiency levels. Advanced learners (mean score for Linguistic Complexity: 4.30) and those aged 28–35 (mean for Cultural Immersion: 4.20) demonstrated a heightened appreciation for deep structural knowledge and authentic cultural exposure. In contrast, younger participants (aged 18–22) prioritized Technological Integration (mean: 4.22) and Learner Satisfaction (mean: 4.18), underscoring generational shifts in engagement styles and expectations. These findings align closely with the FAHP weight distributions, reinforcing the model's capacity to reflect real-world educational dynamics. Sensitivity analysis further confirmed the framework's robustness. Adjusting weights for major criteria by ±30% produced only marginal changes in similarity scores. It did not alter the overall ranking order of the strategies, indicating the model's stability under varying evaluative conditions.

The results support the effectiveness of fuzzy multi-criteria decision-making in educational research, particularly in contexts with high learner diversity and instructional variability. The proposed model offers a scalable, replicable tool for institutions and educators aiming to tailor language instruction effectively. Future research could extend this framework to other linguistic contexts or incorporate longitudinal data to explore how the effectiveness of strategies evolves.

## Data Availability

The original contributions presented in the study are included in the article/supplementary material, further inquiries can be directed to the corresponding author.

## References

[B1] Aarthi K. Narayanamoorthy S. Devi N. S. K. Suvitha K. Almakayeel N. Dincer H. Yuksel S. Kang D. (2026). Spherical bipolar fuzzy decision model for green infrastructure selection. Sci. Rep. 16:12135. doi: 10.1038/s41598-026-41794-8 41781478 PMC13076613

[B2] Abujaber A. A. Abd-Alrazag, A. Al-Qudimat A. R. Nashwan A. J. (2023). A strengths, weaknesses, opportunities, and threats (SWOT) analysis of ChatGPT integration in nursing education: a narrative review. Cureus 15, 1–9. doi: 10.7759/cureus.48643 38090452 PMC10711690

[B3] Alamri H. A. Watson S. Watson W. (2021). Learning technology models that support personalization within blended learning environments in higher education. TechTrends 65, 62–78. doi: 10.1007/s11528-020-00530-3

[B4] Bower K. (2019). Explaining motivation in language learning: a framework for evaluation and research. Lang. Learn. J. 47, 558–574. doi: 10.1080/09571736.2017.1321035

[B5] Budiman B. Ishak J. I. P. Rohani R. Lalu L. M. H. Jaelani S. R. Jaelani M. P. (2023). Enhancing English language proficiency: strategies for improving student skills. J. Sci. Res. Educ. Technol. 2, 1118–1123. doi: 10.58526/jsret.v2i3.205

[B6] Chapelle C. A. (2009). The relationship between second language acquisition theory and computer-assisted language learning. Mod. Lang. J. 93, 741–753. doi: 10.1111/j.1540-4781.2009.00970.x

[B7] Csapó B. Molnár G. (2019). Online diagnostic assessment in support of personalized teaching and learning: the eDia system. Front. Psychol. 10:1522. doi: 10.3389/fpsyg.2019.01522 31333546 PMC6617473

[B8] Dizon G. (2021). Subscription video streaming for informal foreign language learning: Japanese EFL students' practices and perceptions. Tesol J. 12:e566. doi: 10.1002/tesj.566

[B9] Dörnyei Z. (2014). The Psychology of the Language Learner: Individual Differences in Second Language Acquisition. New York, NY: Routledge. doi: 10.4324/9781410613349

[B10] Dörnyei Z. Henry A. (2022). “Accounting for long-term motivation and sustained motivated learning: motivational currents, self-concordant vision, and persistence in language learning,” in Advances in Motivation Science, ed. A. J. Elliot (Oxford: Elsevier), 89–134. doi: 10.1016/bs.adms.2021.12.003

[B11] Ekinci Y. Orbay B. Z. Karadayi M. A. (2022). An MCDM-based game-theoretic approach for strategy selection in higher education. Socioecon. Plann. Sci. 81:101186. doi: 10.1016/j.seps.2021.101186

[B12] Ellis R. (2004). “Individual differences in second language learning,” in The Handbook of Applied Linguistics, eds. A. Davies, and C. Elder (Oxford: Blackwell Publishing Ltd.), 525–551. doi: 10.1002/9780470757000.ch21

[B13] Ellis R. (2015). Understanding Second Language Acquisition, 2nd Edn. Oxford: Oxford University Press.

[B14] Feng C. Cao, L. Wu D. Zhang E. Wang T. Jiang X. . (2025). Acoustic inspired brain-to-sentence decoder for logosyllabic language. Cyborg Bionic Syst. 6:0257. doi: 10.34133/cbsystems.0257 40302941 PMC12038182

[B15] Goh M. Akiba H. Yonezawa Y. Hirai T. Horie M. (2025). Developing intercultural competence through internationalizing higher education: case studies of three Japanese universities. J. Contemp. East Asia Stud. 1–29. doi: 10.1080/24761028.2025.2469950

[B16] Huang C. L. Luo Y.-F. Yang S. C. Lu C. M. (2020). Influence of students' learning style, sense of presence, and cognitive load on learning outcomes in an immersive virtual reality learning environment. J. Comput. Res. 58, 596–615. doi: 10.1177/0735633119867422

[B17] Islam A. (2024). Foreign language acquisition: a study on how people build the motivation to acquire Korean and Japanese languages through media in Bangladesh.

[B18] Japan F. (2020). Survey Report on Japanese-Language Education Abroad 2015. Tokyo: The Japan Foundation Tokyo.

[B19] Kanno Y. (2008). Language and Education in Japan. Hampshire: Palgrave Macmillan. doi: 10.1057/9780230591585

[B20] Kayashima N. Kuroda K. Kitamura Y. (2022). “Japan's international cooperation in education: an overview,” in Japan's International Cooperation in Education: History and Prospects, eds. N. Kayashima, K. Kuroda, and Y. Kitamura (Singapore: Springer), 1–26. doi: 10.1007/978-981-16-6815-9_1

[B21] Li Z. (2023). The development and validation of a scale for measuring L2 learning experience: the underappreciated element of the L2 motivational self system. J. Multiling. Multicult. Dev. 46, 1–20. doi: 10.1080/01434632.2023.2183961

[B22] Liu X. Zhang Z. Zhang Z. (2026). Talking in generalities or talking in detail? the role of unconfirmed customer expectations in reviewers' online expression. Curr. Psychol. 45:177. doi: 10.1007/s12144-025-08741-8

[B23] Long M. (2014). Second *Language Acquisition and Task-Based Language Teaching*. Hoboken, NJ: John Wiley and Sons.

[B24] Newton J. M. Nation I. S. P. (2020). Teaching ESL/EFL Listening and Speaking. New York, NY: Routledge. doi: 10.4324/9780429203114

[B25] Norris J. M. Ortega L. (2000). Effectiveness of L2 instruction: a research synthesis and quantitative meta-analysis. Lang. Learn. 50, 417–528. doi: 10.1111/0023-8333.00136

[B26] Ota H. (2018). Internationalization of higher education: global trends and Japan's challenges. Educ. Stud. Jpn. 12, 91–105. doi: 10.7571/esjkyoiku.12.91

[B27] Richards J. C. Rodgers T. S. (2014). Approaches and Methods in Language Teaching. Cambridge: Cambridge University Press. doi: 10.1017/9781009024532

[B28] Robertson W. C. (2020). Scripting Japan: Orthography, Variation, and the Creation of Meaning in Written Japanese. London: Routledge. doi: 10.4324/9780429331008

[B29] Rossides N. (2025). Exploring Japanese Culture: Not Inscrutable After All. Leicestershire: Troubador Publishing Ltd.

[B30] Sakamoto F. Roger P. (2023). Global competence and foreign language education in Japan. J. Stud. Int. Educ. 27, 719–740. doi: 10.1177/10283153221076905

[B31] Saville-Troike M. Barto K. (2016). Introducing Second Language Acquisition. Cambridge: Cambridge University Press. doi: 10.1017/9781316569832

[B32] Shao S. Tian Y. Zhang Zhang, X. (2025). Knowledge learning-based dimensionality reduction for solving large-scale sparse multiobjective optimization problems. IEEE Trans. Cybern. 55, 3471–3484. doi: 10.1109/TCYB.2025.3558354 40249685

[B33] Sun M. Xiong K. Chen Y. S. Liang Q. Kanjanapathy M. (2025). The operation and efficiency of strategic human resource management in universities of China. J. Chin. Hum. Resour. Manag. 16, 69–83. doi: 10.47297/wspchrmWSP2040-800504.20251604

[B34] Tu Y. Huang C. Pan Y. Hwang G.-J. (2026). Mapping the structure of student burnout in online learning: an integrated Gaussian model and directed acyclic graph approach. Internet High. Educ. 70:101077. doi: 10.1016/j.iheduc.2026.101077

[B35] Wang Y. Zhang Z. Wang C. Li Z. Law R. Zhang Z. (2026). Too concise to decide? the impact of AI-generated review summaries on tourist decision satisfaction. Tour. Manag. 115:105415. doi: 10.1016/j.tourman.2026.105415

[B36] Zhang B. Sang H. Meng L. Jiang X. Lu C. (2025). Knowledge-and data-driven hybrid method for lot streaming scheduling in hybrid flowshop with dynamic order arrivals. Comput. Oper. Res. 184:107244. doi: 10.1016/j.cor.2025.107244

[B37] Zhang H. Tian M. (2024). Unpacking the multi-dimensional nature of teacher competencies: a systematic review. Scand. J. Educ. Res. 69, 1–22. doi: 10.1080/00313831.2024.2369867

[B38] Zhang R. Chen Y. (2026). What can multi-factors contribute to Chinese EFL learners' implicit L2 knowledge? Int. Rev. Appl. Linguist. Lang. Teach. 64, 173–195. doi: 10.1515/iral-2024-0021

[B39] Zhu J. Chen Z. Meo P. D. Guan J. Han Z. Shi W. (2026). KnowPath: an LLM-supported knowledge graph construction and path finding framework to explainable MOOC recommendations. ACM Trans. Inf. Syst. 44, 1–28. doi: 10.1145/3779436

